# A Comparison of the Nutritional Status of Adolescents from Selected Schools of South India and UAE: A Cross-sectional Study

**DOI:** 10.4103/0970-0218.51230

**Published:** 2009-04

**Authors:** Ghalib J Haboubi, Rizwana B Shaikh

**Affiliations:** Department of Community Medicine, Gulf Medical College, Ajman, UAE

**Keywords:** Adolescents, BMI, stunting, thinning

## Abstract

**Objective:**

To assess the nutritional status of adolescents of Indian origin living in India and the United Arab Emirates to see how variable the prevalence is of stunting and wasting among adolescents of the same ethnic background living in different socio-economic and demographic environments.

**Materials and Methods:**

A cross-sectional survey.

**Setting:**

Schools in South India and the United Arab Emirates.

**Participants:**

A total of 2459 adolescent boys and girls between the ages of 10 and 16 years old.

**Results and Discussion:**

Anthropometric measurements from 2459 adolescents between the ages of 10-16 years old, 1200 from India and 1259 from UAE, were collected. The subjects were divided into six age groups with 1-year intervals. Adolescents falling below the age and gender-specific 5^th^ percentile and 3^rd^ percentile of the WHO recommended standards were defined as having thinness and stunting accordingly. Regardless of gender, the rate of stunting was higher in Indian adolescents from India (25.5-51%) when compared with Indian adolescents in UAE (3.1-21%). Thinness was also more in those in India (42-75.4%). When compared with adolescents living in the UAE (4.5-14.4%). The study was done in two groups having a common ethnicity but living in different socio-economic environments. With the results of this study, we can say that improved economic conditions favor better expression of genetic potential for physical growth.

## Introduction

Adolescents are those between the ages of 10 and 19 years old(1) and adolescence is a transitional phase between childhood and adulthood characterized by marked acceleration in growth(2,3) This period is known to be a second chance for growth or catch-up growth for those children who have experienced a nutritional deficit in their early life.(4,5) It is a period of increased nutritional requirements because it is during this time that they gain up to 50% of their adult height and skeletal mass.(1) Since they are relatively healthy compared with other life cycle groups, they have received low priority.(3,5,6) Adolescence is a phase that poses specific challenges for treating diseases and promoting health.(7) Recent reports of the World Health Organization (WHO) suggest that in South East Asian Region a large number of adolescents, who constitute 20% of the population in these countries, suffer from malnutrition and anemia, which adversely impacts their health and development,(2) and that anthropometry is a good indicator of nutritional status and health risks in this group.(1,8) Several factors affect the nutritional status of adolescents. Among these, socio-economic and demographic factors are associated with worldwide patterns of stunting and thinness. It has also been seen that the variability among nations and regions was substantial in comparison with variability among individuals within provinces.(9)

This study was conducted with the objective of assessing the nutritional status of adolescents of Indian origin living in India and the UAE using simple anthropometric measurements and seeing how variable the prevalence is of stunting and wasting among adolescents of the same ethnic background living in different socio-economic and demographic environments.

## Materials and Methods

### Study design

This study was carried out between August and October 2005 in five different schools in South India and one Indian school in Dubai, United Arab Emirates. Two final MBBS students who went to India for the summer training program collected the data from the 5 schools in South India (3 schools in Kerala and 2 schools in Tamil Nadu) and two students collected the data from one Indian school in Dubai. Both the teams were under the constant supervision of the researchers. The data was collected after written informed consent was obtained from the respective school principals. The selection criterion was students of South Indian origin in the age group of 10-16 years old. The sampling design was a non probability consecutive sampling.

#### Measurements

The date of birth of students was obtained from the school health records for their exact age. Their height was measured to the nearest 0.1 centimeter using a calibrated ruler fixed to the wall as the child stood barefoot with the heels, back, and head touching the wall and head held in Frankfurt plane. A thin, wooden scale was placed above the head perpendicular to the ruler and parallel to the ground.(1) Weight was measured to the nearest 0.1 kilogram using a portable weighing machine, which was standardized regularly by calibrating it to zero before each measurement, the child being barefoot, emptied his/her pockets while standing on the weighing machine. Body Mass Index (BMI) was computed using the standard equation: BMI (kg/m^2^) = Weight (kg)/Height^2^ (m^2^). For anthropometric indicators, we followed the WHO expert committee recommendations for adolescents.(1) The cutoff value for stunting was the <3^rd^ percentile of the National Center for Health Statistics (NCHS) standards and thinness was the <5^th^ percentile of NCHS standards. Weight for age was considered to be uninformative and was therefore not included in the analysis. These were calculated for boys and girls separately for the different age groups. Data was analyzed using the SPSS 9.0 software for Windows^™^.

## Results

Data was collected from a total of 2,459 children of South Indian origin of which 1200 were from India and 1259 were from the UAE between the age group of 10-16 years old studying in grades 5 through 10. There were only 96 children in the 10-year age group and, since the number of children of either gender from both the countries was less than 30 in this group, they were excluded from the final analysis. The final analysis included 2,363 children between the age groups of 11-16 years old of which 1,185 were from India and 1,175 were from the UAE. The age and gender distribution of the adolescents is given in [Table 1].

**Table 1:** Age and gender distribution of the study population

Age (years)	India		UAE		Total
			
	Male	Female	Male	Female	
11	97	98	119	104	418
12	112	97	96	102	407
13	104	112	102	108	426
14	101	100	101	102	404
15	110	121	134	110	475
16	65	68	58	42	233
Total	589	596	610	568	2363

The mean and standard deviations of the anthropometric characteristics such as height and BMI by age of the boys and girls from India and the UAE are presented in [Tables 2–5]. Combining all ages, the mean height and BMI of the students from India and the UAE is as follows: the mean height for girls in India was 145.7±10 cms and for the UAE was 151.3±9 cms; for boys in India, it was 146.9±12.4 cms and for the UAE it was 154.8±11.5 cms. The mean BMI for girls in India was 15.8±2.7 and in the UAE was 20.5+4. The mean BMI for boys in India was 15.54±2.4 and for the UAE was 20.2±2.2 cms. An independent sample T test showed that the mean height and BMI was significantly higher in boys and girls from the UAE. The maximum difference is observed in the mean height of boys where we can see that the Indian boys are lagging by almost 8 centimeters behind their UAE counterparts

**Table 2:** Prevalence of stunting in boys in India and the UAE

Age (years)	India			UAE			T test
			
	N	Mean and SD of height (cm)	Percent of students in the <3^nd^ percentile of WHO standards	N	Mean and SD of height (cm)	Percent of students in the <3^rd^ percentile of WHO standards	
11	97	133.15 ± 7.7	48.5	119	142.64±6.3	5.9	Sig
12	112	137.84±6.6	37.5	96	147.2±7.0	3.1	Sig
13	104	145.66±8.99	50	102	153.5±7.8	5.9	Sig
14	101	151.1±7.8	35.8	101	159.4±9.5	12.9	Sig
15	110	157.6±5.6	25.5	134	164.1±8.7	6.0	Sig
16	65	152.5±5.7	36.9	58	165.2±7.3	22.4	Sig
Total	589	146.9±12.4	38.8	610	154.8±11.5	8.19	Sig

SD = Standard deviation, Sig = *P*<0.05

**Table 3:** Prevalence of stunting among girls in India and the UAE

Age	India			UAE			
		
	N	Mean ± SD	Percent of students in the <3^rd^ percentile of WHO standards	N	Mean and SD	Percent of students in the <3^rd^ percentile of WHO standards	T test
11	98	134.2±7.4	29.6	104	140.3±7.3	8.7	Sig
12	97	140±9.2	37.1	102	148±7	10.8	Sig
13	112	145.9±8.5	37.5	108	152.2±6.5	12.2	Sig
14	100	149.0±7.4	51	102	154.9±6.4	15.7	Sig
15	121	152.8±6	33.1	110	157.6±5.6	9.1	Sig
16	68	152.5±5.7	32.4	42	158.6±4.7	4.8	Sig
Total	596	145.7±10	36.9	568	151.3±9	11.6	Sig

SD = Standard deviation, Sig = *P*<0.05

**Table 4:** Prevalence of thinness for boys in India and the UAE

Age	India			UAE			
		
	N	Mean ± SD	Percent of students in the <5^th^ percentile of WHO standards	N	Mean and SD	Percent of students in the <5^th^ percentile of WHO standards	T test
11	97	14.19±2.02	70.1	119	18.80±3.61	7.6	Sig
12	112	14.57±2.21	74.1	96	19.01±3.49	14.6	Sig
13	104	15.43±2.93	61.5	102	20.33±3.91	9.8	Sig
14	101	16.67±2.61	48.5	101	20.69±5	10.9	Sig
15	110	16.21±2.13	61.8	134	21.47±4.51	4.5	Sig
16	65	16.28±2.2	75.4	58	21.21±4.19	17.2	Sig
Total	589	15.54±2.4	64.6	610	20.22±4.2	7.3	Sig

SD = Standard deviation, Sig = *P*<0.05

**Table 5:** Prevalence of thinness for girls in India and the UAE

	India			UAE			
		
Age	N	Mean ± SD	Percent of students in the <5^th^ percentile of WHO standards	N	Mean and SD	Percent of students in the <5^th^ percentile of WHO standards	Ttest
11	98	14.08±1.7	61.2	104	17.77±3.36	14.4	Sig
12	97	15.08±2.64	52.6	102	20.05±5.47	4.9	Sig
13	112	15.89±2.88	43.8	108	19.95±3.81	5.9	Sig
14	100	16.57±2.68	42	102	21.47±4.3	6.9	Sig
15	121	16.57±2.8	52.9	110	22.31±3.96	5.5	Sig
16	68	17.31±2 71	48.5	42	23.28±5 25	11.9	Sig
Total	596	15.88±2.7	50.1	568	20.54±4.4	8.25	Sig
							

SD = Standard deviation, Sig = *P*<0.05

There is a positive linear increasing trend in the mean height for boys and girls between ages 11-15 years old from both India and the UAE with an increment of about 3-8 centimeters every year, not much change is seen from year 15 to 16. The prevalence of stunting among both boys and girls is more in the Indian adolescents in India when compared to their counter parts in the UAE. An independent sample T test showed that there is a significant difference between the 2 groups in both genders and all the age groups. The prevalence of stunting drops at around 15 years for boys, most likely due to the pubertal growth spurt at this age. It is interesting to note that at the commencement of adolescence, adolescents in the UAE are ahead of Indians in both genders by at least 10 cm for boys and 6 cm for girls. The mean height was slightly more for girls than for boys in the various age groups among Indian school children, whereas in the UAE, the mean height for boys was more.

## Discussion

As per the NCHS norms, the prevalence of thinness is more marked in Indian adolescents especially among boys. They have a lower BMI when compared with girls. This is comparable to the observations of Onis, *et al*.(12) and Venkaiah, *et al*.(13) Thinness is more prevalent in the age group of 15-16 years old. This could be because of the growth spurt and sudden increase in height in this age group. The prevalence of thinness is more in Indian girls compared with their counterparts in the UAE, and the overall prevalence of thinness is lower in girls when compared with boys.

Compares the present study with other recent studies conducted in the region. The prevalence of stunting and thinning among South Indian children in India in this study is similar to the students in Bombay,(10) rural north India,(3) and West Bengal.(11)

This study revealed that the prevalence of stunting and thinning was very high among the South Indian students in India when compared with South Indian students in the UAE. This group entered adolescence with poor nutritional status. They lagged behind the students in UAE by 6-10 cm in height and a significant lag in BMI was also noticed. This difference will hamper the catch-up growth and final adult size. Poor nutritional status of girls, especially, has important implications in terms of physical work capacity and adverse reproductive outcomes.(1,15)

This difference can be attributed to the fact that an Indian working in UAE can bring his family into the country only if he earns more than 4000 DH (Rs 50,0000)(15) and subsequently the purchasing power of this family is higher than an average Indian family. The GDP per capita of UAE is (International Dollar 2004) 18,754(16) as compared with India at (International Dollar 2004) 1,830.(17)

The study conducted by De Onis, *et al*.(12) concluded that the NCHS reference data seemed inadequate, and consideration should be given to developing reference data on different ethnic groups. In this study, we can see that the NCHS standards seem to be an adequate reference data for international use because the two communities in this study are of the same ethnicity i.e., South Indians but in different socio-economic and demographic environments. Growth is influenced by both genetic and environmental factors. If the socio-economic status is improved, there is full expression of the genetic potential and the growth rates are comparable to the western standards.(2) Given the results of the study, we can say that improved economic conditions favor better expression of genetic potential for physical growth.

It is also seen that food security at the national level, as measured by the supply of food energy, has a strong link to both stunting and wasting. Whether or not children are malnourished is as much a consequence of factors at the national level as it a consequence of individual household circumstances.(9)

The results show that there is a need for intervention not only at the household level but also at the national level to bring about some real change. The health sector should play a role in integrating adolescent nutrition in other programs.(5) Our recommendation is to make it the responsibility of the state by having mandatory mid-day meal programs both in public and private schools, because today's adolescents are the future of the country.
